# Association between life’s essential 8 and overactive bladder

**DOI:** 10.1038/s41598-024-62842-1

**Published:** 2024-05-23

**Authors:** Guoliang Feng, Shaoqun Huang, Weimin Zhao, Hongyang Gong

**Affiliations:** 1 Department of Oncology, Nanchang Third Hospital, Nanchang, Jiangxi China; 2 Department of Oncology Surgery, Fuzhou Traditional Chinese Medicine Hospital, Fuzhou City, Fujian Province China; 3 Department of Clinical Medicine, School of Medicine, Shihezi University, Shihezi City, China; 4 Department of Physiology, College of Medicine, Chosun University, 309 Pilmun-daero, Dong-gu, Gwangju, 61452 Republic of Korea

**Keywords:** Overactive bladder, Cardiovascular health, Life’s essential 8, NHANES, Medical research, Nephrology, Risk factors

## Abstract

Limited research has explored the relationship between overactive bladder (OAB) and cardiovascular health (CVH). We aim to examine the correlation between OAB and CVH in American adults, utilizing the Life’s Essential 8 (LE8). We included 70,190 individuals from the National Health and Nutrition Examination Survey spanning from 2005 to 2018. In our study, the independent variable is LE8 score, where higher scores denote better CVH. The dependent variable is the presence of OAB. We employed multivariable logistic regression along with restricted cubic splines to evaluate the association between LE8 and OAB. Additionally, we performed interaction analyzes on subgroups to validate the findings. There is a significant negative correlation between LE8 scores and OAB. Upon adjusting for all covariates, a 10-point increase in LE8 total score correlated with a 17% decrease in the odds of OAB [0.83 (95% CI 0.78, 0.89)]. Compared to participants with lower LE8 scores, those with higher LE8 scores had a 46% lower probability of developing OAB. Consistent results were also observed in the association between scores of four health behaviors and four health factors and OAB. Furthermore, a notable interaction was observed between LE8 scores and smoking status. This study showed a significant negative correlation between LE8 scores and OAB prevalence.

## Introduction

Overactive bladder (OAB) manifests as urinary urgency, often accompanied by nocturia and increased urinary frequency, with or without the presence of urge urinary incontinence^1^. Concurrently, OAB is considered a highly prevalent, bothersome, and distressing condition^2^. Furthermore, besides significantly impacting quality of life, OAB evidently imposes substantial global economic burdens on healthcare and social support systems. The estimated costs of urge urinary incontinence (UUI) due to OAB in the United States reached $65.9 billion in 2007, $76.2 billion in 2015, and $82.6 billion in 2020^3^. However, the risk factors and pathogenesis of OAB remain incompletely understood. Several studies indicate that OAB might correlate with various risk factors, including BMI^4^, diet^5^, nicotine^6^, drinking^7^, daily exercise^8^, sleep disturbances^9^, diabetes^10^, hypertension^11^, and dyslipidemia^12^.

In 2022, the American Heart Association (AHA) introduced the Life’s Essential 8 (LE8) score, an enhanced quantitative algorithm used to assess cardiovascular health (CVH)^13^. In contrast to the Life’s Simple 7 introduced by the AHA in 2010, the LE8 scoring system, introduced in 2022, demonstrates greater sensitivity to individual differences and underscores the importance of preserving or enhancing cardiovascular health^14^. LE8 scores are computed utilizing four health behaviors (sleep, smoking, daily exercise, and diet) along with four health factors (BMI, non-HDL-C, blood sugar, and blood pressure)^15^.

Although extensive research has been conducted on the individual effects of the four health behaviors and four health factors on OAB, there has been no investigation into the relationship between LE8 (a composite index of these factors) and OAB. Therefore, we utilized a large population dataset from the NHANES covering the period from 2005 to 2018 to conduct a cross-sectional analysis to explore the correlation between LE8 and OAB. These findings may offer new strategies for prevention and management of OAB in clinical practice.

## Methods

### Data source

The National Health and Nutrition Examination Survey (NHANES) is conducted by the National Center for Health Statistics (NCHS) to gather demographic data on the health and nutrient consumption of American citizens. The study protocols have received ethical approval from the NCHS Research Ethics Review Board, in accordance with the Declaration of Helsinki. Written informed consent was obtained from all adult participants. Our secondary analysis adheres to the STROBE guidelines for cross-sectional studies^16^, obviating the need for additional institutional review board approval^17^. Detailed information on NHANES’ methodology and ethical considerations can be accessed on the CDC and NCHS website (https://www.cdc.gov/nchs/nhanes/index.htm).

### Study participants

In this cross-sectional study, nationally representative data from the National Health and Nutrition Examination Survey (NHANES) were utilized. Among the 70,190 participants across 7 NHANES cycles spanning from 2005 to 2018, there were 39,749 participants aged ≥ 20 years. After excluding participants with missing data on CVH indicators (n = 12,763) and OAB indicators (n = 1136) from the total of 39,749 participants, a final cohort of 25,850 participants was included in the study (see Supplementary Fig. 1).

### Cardiovascular health evaluation (exposure)

CVH is evaluated through the LE8 score, where higher scores signify superior CVH. It incorporates eight key elements: four health behaviors (sleep, smoking, daily exercise, and diet) and four health factors (BMI, non-HDL-C, blood sugar, and blood pressure). Detailed descriptions of calculating scores for each LE8 indicator using NHANES data can be found in Supplementary Table 1. Briefly, the scores for each of the eight CVH indicators range from 0 to 100. The overall LE8 score is determined by averaging the scores for the eight individual factors. According to previous research, LE8 scores falling within the ranges of 80–100, 50–79, and 0–49 correspond to high, moderate, and low levels of CVH, respectively^15^. Dietary components were evaluated using the Healthy eating index-2015 (HEI-2015). The components and scoring criteria of HEI-2015 are outlined in Supplementary Table 2. Dietary intake of participants collected from two 24-h dietary recalls was combined with food pattern equivalent data from the United States Department of Agriculture (USDA) to construct and calculate HEI-2015 scores^18^. Sleep duration, smoking, daily exercise, medication, and history of diabetes were obtained from standardized questionnaires.

### OAB assessment (outcome)

In patients, it is essential to consider urgency urinary incontinence and nocturia as indicative of OAB according to its definition. We utilized the following three questions from NHANES questionnaires KIQ044, KIQ450, and KIQ480 to assess urinary incontinence and nocturia^19^: (1) Within the last year, have you experienced involuntary urine leakage accompanied by a sense of urgency or pressure, and were unable to reach the toilet promptly? (2) How frequently does this happen? (3) Over the last month, how often do you usually wake up to urinate between going to bed at night and getting up in the morning?

Subsequently, we used the Overactive Bladder Symptom Score (OABSS) questionnaire to quantify OAB^20^. The detailed scoring criteria can be found in Supplementary Table 3. Based on previous research^21^, the OABSS score for each participant was obtained by summing the scores for urgency urinary incontinence and nocturia. In this investigation, individuals with a total score of 3 or higher were regarded as having overactive bladder^21^.

### Covariables

Based on previous studies^1,22^, the study covariates include age, gender, race, marital status, education level, household poverty income ratio PIR, obesity, smoking, drinking, sleep disturbances, hypertension, diabetes, and self-reported hyperlipidemia. For detailed information regarding these covariates, please refer to Supplementary Table 4.

### Statistical analysis

Sampling weights were applied in all statistical analyses to ensure that estimates were representative of the national population. Continuous variables are expressed as mean ± SD, whereas categorical variables are depicted as frequencies (percentages). Weighted t-tests were used to compare continuous variables between different LE8 groups, and weighted chi-square tests were used for categorical variables. Weighted logistic regression was utilized to explore the correlation between LE8 scores and OAB. Three logistic regression models were developed: Model 1 remained unadjusted for potential confounders. Model 2 was fine-tuned considering age, gender, race, marital status, education level, and PIR as covariates. Model 3 was additionally refined by incorporating sleep disruptions, obesity, smoking habits, alcohol intake, hypertension, diabetes, and hyperlipidemia as further adjusting factors. Furthermore, in Model 3, we considered the LE8 score as a continuous variable and applied RCS to elucidate the association between LE8 scores and the risk of OAB. Subsequently, subgroup analyzes were performed stratified by covariates in Model 3. Interaction analyses were then carried out to explore potential variations in associations between subgroups. Statistical analyses were performed using R software (version 4.3.1). Statistical significance was defined as a two-sided P-value less than 0.05.

### Ethics approval and consent to participate

This study was reviewed and approved by the NCHS Ethics Review Board. The patients/participants provided written informed consent to participate in this study.

## Results

### Baseline characteristics

**Figure 1:**
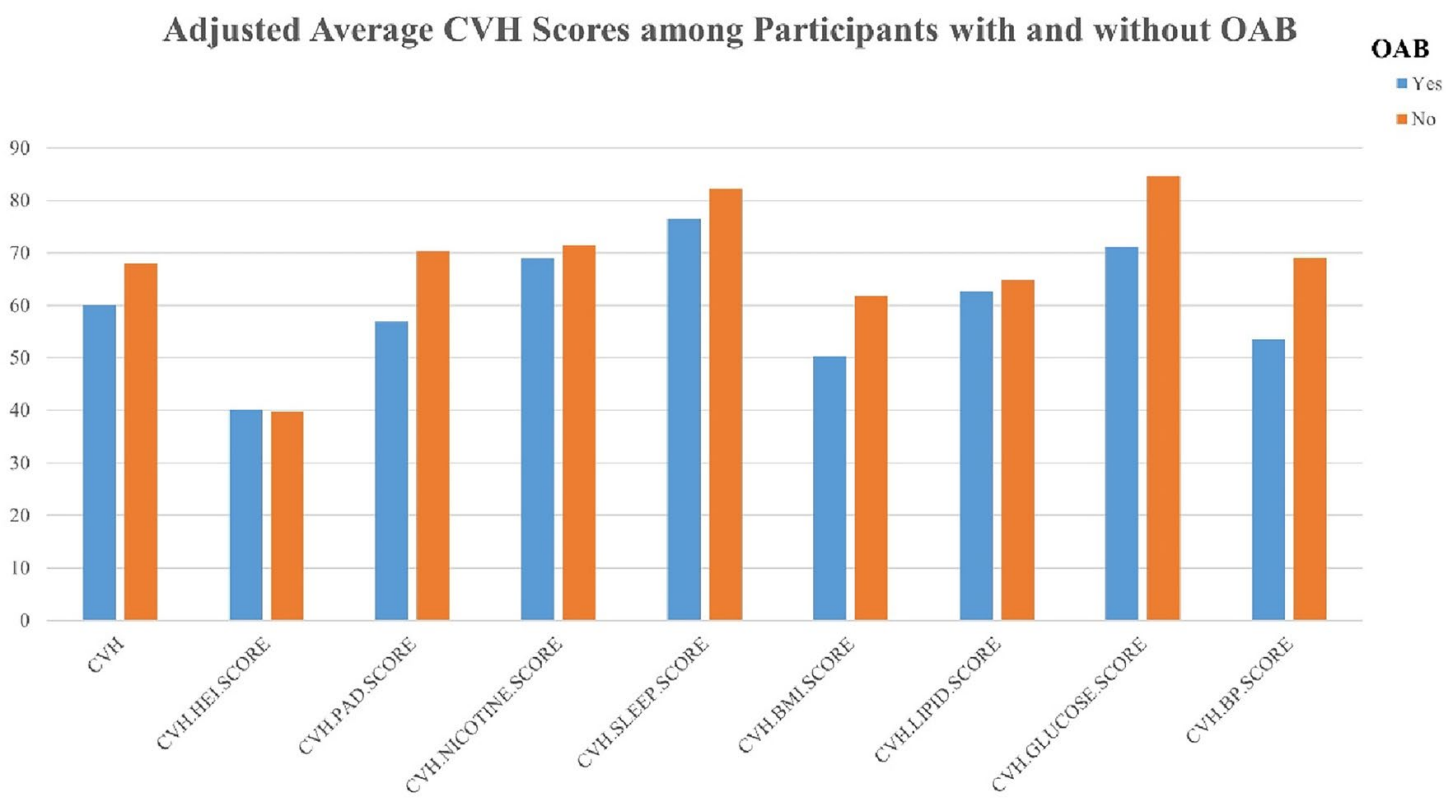
Distribution of Life’s Essential 8 total score and 8 sub-scores in participants with and without overactive bladder. Abbreviation: OAB, overactive bladder. Note: a higher CVH score indicates better cardiovascular health.

This study encompassed 25,850 participants aged 20 years or above, which extrapolates to roughly 162.17 million U.S. adults. The prevalence of OAB was 15.87% (equivalent to 25.74 million individuals), and the mean (SD) CVH score was 68.25 (14.33). Figure 1 illustrates the distribution of LE8 score and the 8 sub-scores among participants with and without OAB. Participants were classified based on LE8 scores as follows: 10.49% showed low CVH (LE8 < 50), 66.79% had moderate CVH (50 ≤ LE8 < 80), and 21.72% were labeled as high CVH (LE8 ≥ 80). Preliminary assessment indicated that participants who were younger, White, married, of higher socioeconomic status, had healthier lifestyles, and were in better physical condition exhibited higher LE8 scores (LE8 ≥ 80) compared to those in the low CVH group. Additionally, as LE8 scores increased, the prevalence of OAB gradually decreased (31.55%, 16.23%, 7.56%). Further details are provided in Table 1.

**Table 1:** Characteristics of participants at baseline by different levels of CVH estimated by the LE8 score.

Characteristic	Total	Low (LE8 < 50)	Moderate (50 ≤ LE8 < 80)	High (LE8 ≥ 80)
Prevalence, % (weighted N, in millions)	100.00 (162.17)	10.49 (17.02)	66.79 (108.32)	22.72 (36.83)
No. of participants in sample	25,850	3376	17,525	4949
OAB, % (weighted N, in millions)				
No	84.13 (136.43)	68.45(11.64)	83.77 (90.73)	92.44 (34.06)
Yes	15.87 (25.74)	31.55(5.36)	16.23 (17.59)	7.56 (2.79)
Age, % (weighted N, in millions)				
20–40	36.00 (58.37)	17.82 (3.03)	33.41 (36.19)	52.00 (19.16)
41–60	38.64 (62.66)	45.37 (7.72)	39.55 (42.84)	32.84 (12.10)
> 60	25.37 (41.14)	36.81 (6.26)	27.04 (29.29)	15.16 (5.59)
Gender, % (weighted N, in millions)				
Male	78.93 (48.67)	7.89 (46.35)	55.94 (51.65)	15.10 (40.99)
Female	83.24 (51.33)	9.13 (53.65)	52.38 (48.35)	21.73 (59.01)
Race, % (weighted N, in millions)				
Mexican American	7.72 (12.52)	6.89 (1.17)	8.01 (8.68)	7.25 (2.67)
Non-Hispanic White	70.55 (114.42)	67.68 (11.52)	70.34 (76.19)	72.50 (26.71)
Non-Hispanic Black	10.05 (16.30)	15.53 (2.64)	10.57 (11.45)	5.97 (2.21)
Other	11.68 (18.94)	9.89 (1.68)	11.08 (12.00)	14.28 (5.26)
Marital status, % (weighted N, in millions)				
No	35.11 (56.94)	40.74 (6.93)	34.48 (37.35)	34.35 (12.65)
Yes	64.89 (105.23)	59.26 (10.08)	65.52 (70.97)	65.65 (24.18)
Education, % (weighted N, in millions)				
Below high school	14.16 (22.97)	25.61 (4.36)	14.77 (15.99)	7.09 (2.62)
High School or above	85.84 (139.20)	74.39 (12.66)	85.23 (92.32)	92.91 (34.23)
PIR, % (weighted N, in millions)				
Not Poor	80.86 (123.12)	68.76 (10.93)	80.89 (82.15)	86.31 (30.04)
Poor	19.14 (29.14)	31.24 (4.97)	19.11 (19.41)	13.69 (4.76)
Obesity, % (weighted N, in millions)				
No	62.72 (101.71)	24.71 (4.20)	58.39 (63.25)	93.00 (34.26)
Yes	37.28 (60.46)	75.29 (12.81)	41.61 (45.07)	7.00 (2.58)
Smoking, % (weighted N, in millions)				
Never	54.97 (89.15)	24.60 (4.19)	51.25 (55.51)	79.96 (29.45)
Former	25.91 (42.02)	29.80 (5.07)	27.98 (30.31)	18.03 (6.64)
Current	19.11 (31.00)	45.60 (7.76)	20.77 (22.50)	2.01 (0.74)
Drinking, % (weighted N, in millions)				
No	22.05 (35.39)	26.96 (4.56)	21.94 (23.51)	20.11 (7.33)
Yes	77.95 (125.08)	73.04 (12.33)	78.06 (83.65)	79.89 (29.09)
Sleep disorder, % (weighted N, in millions)				
No	84.27 (136.53)	72.10 (12.26)	84.08 (90.99)	90.45 (33.29)
Yes	15.73 (25.48)	27.90 (4.74)	15.92 (17.22)	9.55 (3.51)
Hypertension, % (weighted N, in millions)				
No	61.87 (100.34)	29.49 (5.02)	58.21 (63.05)	87.60 (32.27)
Yes	38.13 (61.83)	70.51 (12.00)	41.79 (45.27)	12.40 (4.57)
Diabetes, % (weighted N, in millions)				
No	78.58 (68.41)	44.25 (4.92)	79.13 (45.59)	97.65 (17.89)
Yes	21.42 (18.65)	55.75 (6.19)	20.87 (12.02)	2.35 (0.43)
High cholesterol, % (weighted N, in millions)				
No	61.90 (89.64)	42.17 (6.66)	59.92 (57.74)	77.28 (25.23)
Yes	38.10 (55.18)	57.83 (9.13)	40.08 (38.63)	22.72 (7.42)
AHA LE8 score (SD)				
Mean total CVH score (mean (SD))	68.25 (14.33)	42.22 (6.33)	66.05 (8.04)	86.75 (5.05)
Mean HEI-2015 diet score (mean (SD))	39.41 (31.32)	20.64 (23.48)	35.41 (29.58)	59.84 (29.38)
Mean physical activity score (mean (SD))	71.83 (41.04)	27.37 (40.89)	71.33 (40.79)	93.84 (18.77)
Mean tobacco exposure score (mean (SD))	71.43 (38.64)	42.52 (42.74)	68.99 (39.34)	91.94 (18.80)
Mean sleep health score (mean (SD))	83.44 (24.21)	66.07 (31.15)	83.01 (23.92)	92.73 (14.95)
Mean body mass index score (mean (SD))	60.48 (33.56)	31.72 (29.06)	56.62 (32.34)	85.12 (21.46)
Mean blood lipid score (mean (SD))	64.34 (30.24)	43.35 (29.12)	61.20 (29.15)	83.28 (23.34)
Mean blood glucose score (mean (SD))	85.89 (24.09)	61.29 (29.84)	85.73 (23.51)	97.72 (9.99)
Mean blood pressure score (mean (SD))	69.21 (30.95)	44.84 (30.13)	66.12 (30.41)	89.53 (19.05)

Mean (SD) for continuous variables: the *P* value was calculated by the weighted linear regression model.

Percentages (weighted N, in millions) for categorical variables: the *P* value was calculated by the weighted chi-square test.

Cardiovascular health (CVH) is categorized into 3 grades, low: LE8 score < 50, medium:50 ≤ LE8 score < 80, high:LE8 score ≥ 80.

*OAB* overactive bladder, *AHA* American Heart Association, *LE8* life’s essential 8, *CVH* cardiovascular health, *PIR* ratio of family income to poverty, *HEI-2015* healthy eating index-2015.

A higher CVH score indicates better cardiovascular health.

### Association of LE8 and OAB

**Figure 2:**
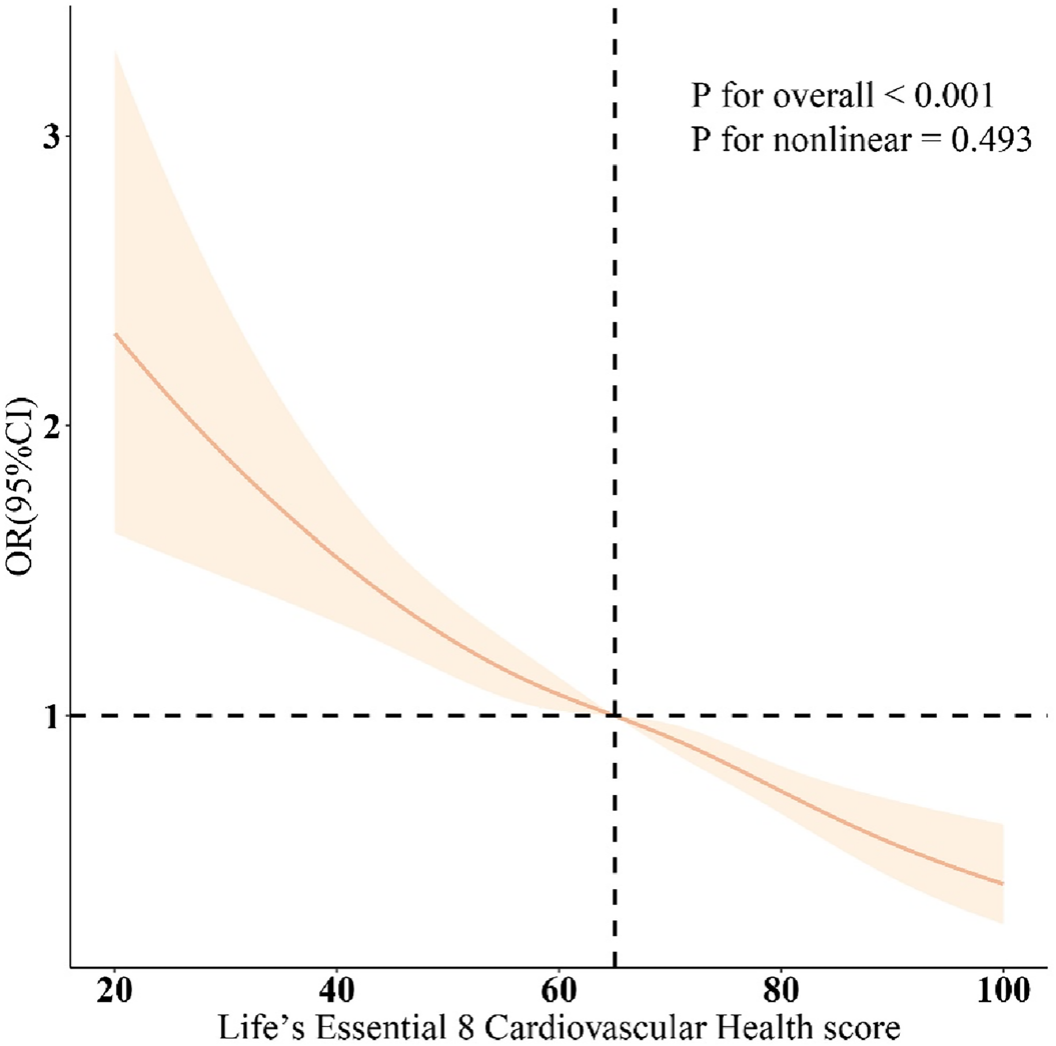
Dose–response relationships between the life’s essential 8 total score and overactive bladder. OR (solid lines) and 95% confidence levels (shaded areas) were adjusted for age, gender, education level, marital, PIR, race, obesity, smoking, drinking, sleep disorder, hypertension, diabetes, and high cholesterol. Note: a higher CVH score indicates better cardiovascular health.

As shown in Table 2, three different models are employed to assess the association between CVH and its eight sub-scores and OAB. After adjusting for all covariates in the model, a 10-point increase in LE8 is linked to a 17% reduction in the odds of OAB [odds ratio: 0.83 (95% confidence interval: 0.78, 0.89)]. Moreover, participants in the high CVH group (LE8 ≥ 80), when compared to those with low CVH (LE8 < 50), show an odds ratio of 0.54 (95% CI 0.39, 0.75) for OAB (*P* < 0.001). Additionally, in the fully adjusted model, except for dietary score, smoking, lipid, and blood pressure scores, all other CVH sub-scores maintain a significant negative correlation with OAB. Figure 2 further illustrates a significant negative association between LE8 and the odds of OAB (overall *P* < 0.001; non-linear *P* = 0.493). In addition, to improve the reliability of this study, we also assessed the relationship between urge incontinence and CVH. (Supplementary Table 5).

**Table 2:** Adjusted odds ratios of life’s essential 8 cardiovascular health (CVH) score and OAB, NHANES 2005–2018.

CVH components (per 10 scores)	Model 1 [OR (95% CI)]	*P*	Model 2 [OR (95% CI)]	*P*	Model 3 [OR (95% CI)]	*P*
Total CVH score	0.67 (0.65, 0.69)	< 0.001	0.75 (0.72, 0.78)	< 0.001	0.83 (0.78, 0.89)	< 0.001
Low (0–49)	1 (ref.)		1 (ref.)		1 (ref.)	
Moderate (50–79)	0.42 (0.37, 0.48)	< 0.001	0.55 (0.48, 0.63)	< 0.001	0.78 (0.65, 0.94)	0.011
High (80–100)	0.18 (0.15, 0.21)	< 0.001	0.30 (0.25, 0.37)	< 0.001	0.54 (0.39, 0.75)	< 0.001
*P for trend*	< 0.001		< 0.001		< 0.001	
Subgroup CVH scores						
HEI diet score	1.00 (0.99, 1.02)	0.800	0.97 (0.95, 0.98)	< 0.001	0.99 (0.96, 1.01)	0.200
Physical activity score	0.93 (0.92, 0.94)	< 0.001	0.96 (0.95, 0.97)	< 0.001	0.97 (0.95, 0.98)	< 0.001
Tobacco exposure score	0.98 (0.97, 0.99)	< 0.001	0.97 (0.96, 0.98)	0.700	1.00 (0.94, 1.06)	> 0.9
Sleep health score	0.92 (0.90, 0.93)	< 0.001	0.92 (0.91, 0.94)	< 0.001	0.95 (0.93, 0.97)	< 0.001
Body mass index score	0.90 (0.89, 0.92)	< 0.001	0.91 (0.90, 0.92)	< 0.001	0.90 (0.86, 0.94)	< 0.001
Blood lipid score	0.96 (0.95, 0.98)	< 0.001	0.99 (0.98, 1.01)	0.300	1.00 (0.97, 1.02)	0.700
Blood glucose score	0.83 (0.82, 0.84)	< 0.001	0.90 (0.88, 0.91)	< 0.001	0.95 (0.91, 0.99)	0.014
Blood pressure score	0.86 (0.85, 0.88)	< 0.001	0.94 (0.92, 0.96)	< 0.001	0.99 (0.96, 1.02)	0.500

Model 1: no covariates were adjusted;

Model 2: age, gender, education level, marital, PIR, and race were adjusted;

Model 3: age, gender, education level, marital, PIR, race, obesity, smoking, drinking, sleep disorder, hypertension, diabetes, and high cholesterol were adjusted;

*OAB* overactive bladder, *AHA* American Heart Association, *LE8* life’s essential 8, *CVH* cardiovascular health, *PIR* ratio of family income to poverty, *HEI-2015* healthy eating index-2015.

A higher CVH score indicates better cardiovascular health.

### Subgroup analyses

**Figure 3:**
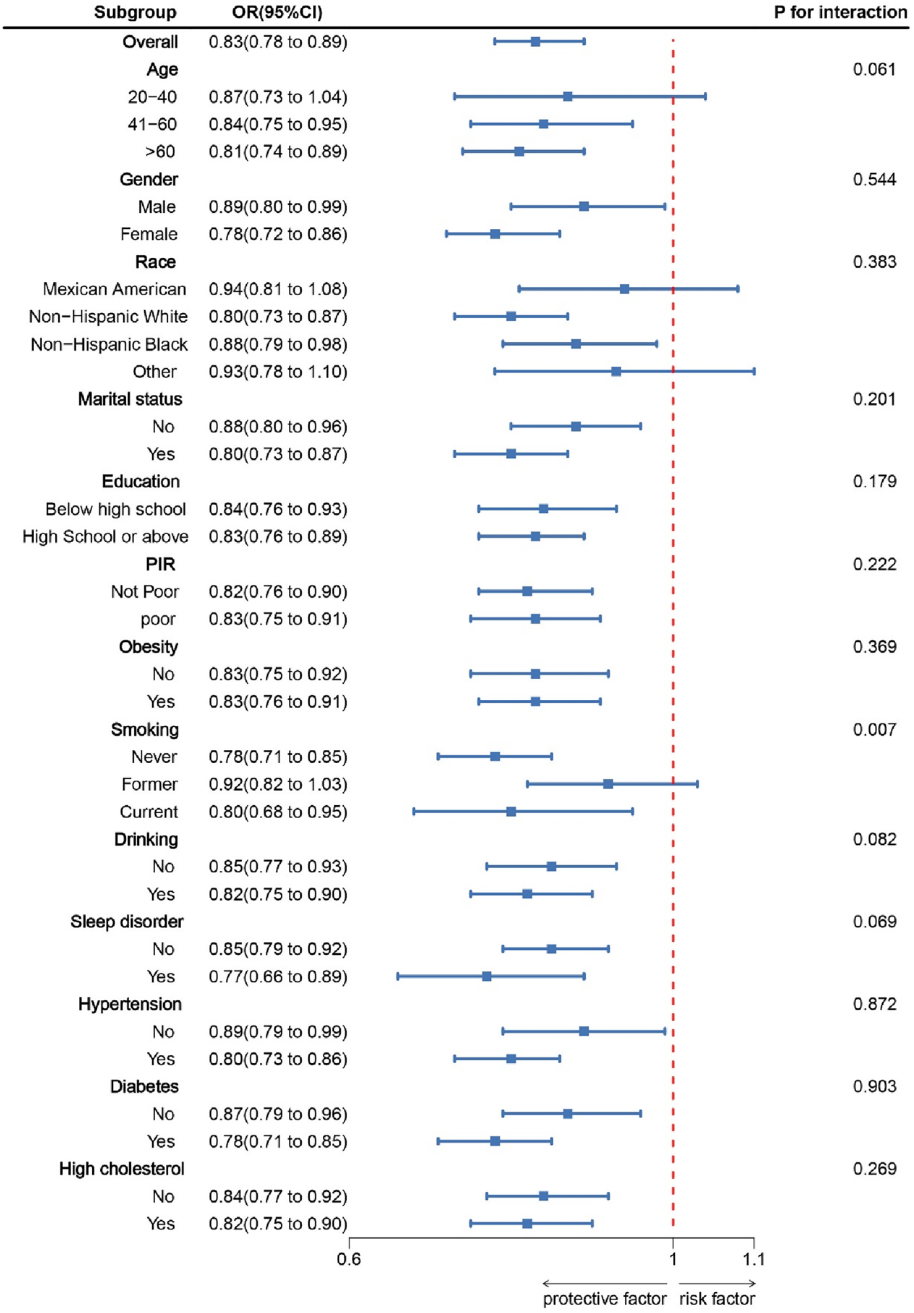
Subgroup analysis between LE8 and overactive bladder. ORs were calculated as per 10 scores increase in LE8. Analyses were adjusted for age, gender, education level, marital, PIR, race, obesity, smoking, drinking, sleep disorder, hypertension, diabetes, and high cholesterol. Note: a higher CVH score indicates better cardiovascular health.

The results of subgroup analysis are depicted in Fig. 3. A negative correlation was observed between LE8 scores of most subgroups and OAB (*P* < 0.05). A significant interaction between LE8 scores and smoking status was observed (*P* < 0.05). With every 10-point increase in the LE8, the odds of OAB decreased by 22% among non-smokers [0.78 (0.71, 0.85)], which was significantly higher than those among former smokers [0.92 (0.82, 1.03)] and current smokers [0.80 (0.68, 0.95)].

## Discussion

In this study, we detected a notable negative association between CVH, as quantified by LE8 scores, and the risk of OAB. Subgroup analysis further revealed a stronger negative correlation between LE8 scores and OAB among non-smokers. These findings highlight the potential influence of cardiovascular health on the occurrence of OAB and underscore the significance of monitoring and lowering OAB incidence through LE8 quantified CVH scores.

To the best of our understanding, this study is the first to examine the association between the novel indicator of CVH, LE8 scores, and the prevalence of OAB. Prior studies have mainly concentrated on the connection between specific cardiovascular diseases and OAB. Research from Turkey suggested a heightened occurrence of coronary artery disease among individuals experiencing OAB symptoms^23^. Another study demonstrated that patients with significantly severe coronary artery stenosis exhibited severe OAB symptoms OR [1.07 (95% CI 1.05, 1.09)]^24^.

However, understanding the association between cardiovascular diseases and OAB alone is insufficient; the pathogenesis of OAB is also closely related to various cardiovascular risk factors. In this study, the CVH scores measured by LE8 encompass common risk factors shared by OAB and cardiovascular risk. For instance, smoking elevates the risk of both OAB and atherosclerosis, exacerbating daytime frequency, nocturia, and urgency^6^. Furthermore, in a cross-sectional study involving 16,978 participants, moderate and poor sleep patterns were associated with a significant 26% and 38% increase in OAB risk, respectively^25^. Concurrently, sleep disorders accelerate the progression of many cardiovascular diseases^26^. Furthermore, metabolic syndrome, which includes obesity, dyslipidemia, abnormal blood glucose, and hypertension, has been demonstrated to play a pivotal role in both OAB and cardiovascular health^27,28^.The evidence above suggests that CVH scores assessed by LE8 hold promise in acting as indicators for both cardiovascular health status and OAB incidence. Our findings illustrate a negative correlation between CVH and OAB from these various angles.

The mechanisms that explain the inverse relationship between cardiovascular health and OAB incidence are multifaceted and intricate. Possible mechanisms include: (1) Vascular pathology: Conditions such as atherosclerosis represent systemic diseases affecting multiple vessels, including coronary and pelvic arteries, with pelvic ischemia closely linked to OAB^29^; (2) Shared pathophysiological mechanisms: Congestive heart failure and OAB are both associated with disorders of neurogenic and autonomic nervous system function^30^; (3) Iatrogenic treatment factors: Diuretics commonly used by cardiac patients may increase the incidence of OAB. These medications may elevate urinary frequency and potentially lead to urgency and incontinence^31^.

It has been shown that bladder ischaemia and oxidative stress are associated with OAB^32^. Specifically, OAB may occur as a result of a disturbed balance between certain pro-oxidants (e.g., free radicals and reactive substances) produced in the body and antioxidant-induced oxidative stress. In addition, abnormal increases in M2/M3 muscarinic receptors and P2X3 purinergic receptors, as well as in cyclooxygenase-2 (COX-2), prostaglandins, and leukotrienes, are also involved in the pathophysiological process of OAB. In contrast, in LE8, nicotine exposure is assessed primarily through smoking habits. Smoking not only enhances oxidative stress through the production of reactive oxygen radicals in the smoke, but also weakens the antioxidant defence systems^33^. In addition, obesity, abnormalities in blood pressure, blood glucose and lipids, as well as sleep disorders, which are constituents of LE8, all influence the aforementioned pathophysiological processes^34–37^. Thus, these studies collectively highlight the important role of LE8 in relation to OAB, providing further theoretical support for the present study provides further theoretical support.

The findings of this study may offer important guidance for the management and prevention of OAB risk. Firstly, this is the first instance of utilizing LE8 to predict the risk of OAB, presenting potential clinical value. Secondly, we accounted for appropriate sampling weights in our analysis to mitigate oversampling bias, rendering our conclusions more reliable. Lastly, based on a nationally representative sample of American adults, these results can be extrapolated to larger populations^38^. Nevertheless, the study is subject to certain limitations: (1) The study’s cross-sectional design hinders the establishment of causality, thereby preventing definitive determination of the causal link between LE8 and OAB; (2) The diagnosis of OAB primarily relies on questionnaire forms, which may introduce measurement errors; (3) Despite adjusting for many other confounders, due to limitations of the NHANES database, we were unable to include a number of potential confounders that were associated with OAB (including factors such as prostatic hypertrophy, neurogenic, muscular, inflammatory, and bladder outlet).

## Conclusion

In summary, our study suggests a negative association between CVH as estimated by LE8 scores and OAB, shedding light on the potential link between OAB and compromised cardiovascular health.

### Supplementary Information

Supplementary Information.

## Data Availability

The datasets used and/or analysed during the current study available from the corresponding author on reasonable request.

## Abbreviations

NHANES: National Health and Nutrition Examination Survey
LE8: Life’s essential 8
OAB: Overactive bladder
AHA: American Heart Association
CVH: Cardiovascular health
HEI-2015: Healthy eating index-2015
